# Randomized phase II study of cetuximab versus irinotecan and cetuximab in patients with chemo-refractory KRAS codon G13D metastatic colorectal cancer (G13D-study)

**DOI:** 10.1007/s00280-016-3203-7

**Published:** 2016-11-22

**Authors:** Masato Nakamura, Toru Aoyama, Keiichiro Ishibashi, Akihito Tsuji, Yasutaka Takinishi, Yoshiaki Shindo, Junichi Sakamoto, Koji Oba, Hideyuki Mishima

**Affiliations:** 1 Aizawa Comprehensive Cancer Center, Aizawa Hospital, Nagano, Japan; 2 Department of Gastrointestinal Surgery, Kanagawa Cancer Center, 2-3-2 Nakao, Asahi-ku, Yokohama, 241-8515 Japan; 3 Department of Digestive Tract and General Surgery, Saitama Medical Center, Saitama Medical University, Kawagoe, Japan; 4 Department of Clinical Oncology, Kagawa University Faculty of Medicine, Kagawa, Japan; 5 Cancer biology, University of Hawaii Cancer Center, Honolulu, USA; 6 Department of Gastrointestinal Surgery, Nakadori General Hospital, Akita, Japan; 7 Department of Surgery, Tokai Central Hospital, Kakamigahara, Japan; 8 Department of Biostatistics, The University of Tokyo, Tokyo, Japan; 9 Cancer Center, Aichi Medical University, Nagakute, Japan

**Keywords:** Cetuximab, KRAS G13D, Colorectal cancer

## Abstract

**Purpose:**

This study investigated the efficacy and safety of cetuximab-based treatment in patients with chemotherapy-resistant refractory mCRC with KRAS G13D mutation.

**Patients and methods:**

An assessment of the efficacy and safety of cetuximab-based treatment was performed in an observation-enriched randomized controlled study comparing the cetuximab alone group (Cet group) and the combination of cetuximab and irinotecan group (CetI group) for KRAS G13D-mutated mCRC in Japan. In this study, the patients received a biweekly (500 mg/m^2^ on day 1) or weekly (250 mg/m^2^) intravenous infusion of cetuximab in Cet group, or a biweekly (500 mg/m^2^ on day 1) or weekly (250 mg/m^2^) intravenous infusion of cetuximab followed by irinotecan (150 mg/m^2^) in CetI group. Propensity score adjustment was used to achieve balance in the observational arm.

**Results:**

Data from a total of 29 patients (10 in Cet group, 19 in CetI group) were analyzed. Crude median progression-free survival time was 2.9 months in the Cet group and 2.5 months in the CetI group. Crude disease control rates were 55.6% in the Cet group and 47.4% in the CetI group. After a median follow-up of 43 months, the crude median overall survival was 8.0 months in the Cet group and 7.6 months in the CetI group. Cetuximab-based treatment did not markedly increase any characteristic toxicity and was generally well tolerated. Propensity score analyses adjusted for performance status and number of metastases showed comparable results with the crude results.

**Conclusion:**

Cetuximab-based treatment seemed to benefit patients with chemotherapy-resistant, refractory KRAS G13D-mutated mCRC. Our results might support the administration of cetuximab-based treatment for KRAS-mutant mCRC and would be able to provide treatment flexibility in this setting.

## Introduction

Colorectal cancer (CRC) is the third-most common malignant disease and one of the most frequent causes of cancer-related death worldwide [1]. Complete resection is essential for the cure of CRC. However, metastatic CRC (mCRC) cannot be cured by surgical resection alone, and the prognosis of such patients is poor [2, 3].

Significant advances in the treatment of mCRC have been achieved by the addition of molecular targeting agents to standard mCRC chemotherapy. Cetuximab is the first chimeric human-mouse monoclonal antibody of immunoglobulin G1 (IgG1) subclass directed against epidermal growth factor receptor (EGFR) [4, 5]. Cetuximab has been shown to be effective in the treatment of mCRC when administered either as monotherapy or in combination with other chemotherapeutic agents [6, 7]. However, retrospective studies and prospective randomized phase III studies of mCRC indicated that mCRC patients with KRAS codon 12 or 13 mutations do not benefit from cetuximab [8]. The health authorities in Europe, the US, and Japan have therefore recommended compulsory KRAS mutation testing [9, 10]. In this respect, patients with KRAS codon 12 or KRAS codon 13 mutated tumors cannot receive cetuximab in Europe or the US. Although standard cytotoxic chemotherapy and/or bevacizumab are typically used for mCRC treatment, no valid and effective treatment for refractory mCRC patients with mutations of KRAS either in codon 12 or 13 has yet been established. In this regard, the development of a treatment for such cases is imperative to improve the outcome of these mCRC patients.

Recently, De Roock et al. [11] investigated the association between KRAS mutation status (G13D vs. other KRAS mutations) and response and survival in a pooled data set of 579 patients with chemotherapy-refractory CRC treated with cetuximab. They found that administration of cetuximab was associated with longer overall and progression-free survival among patients with chemotherapy-refractory CRC with G13D-mutated tumors than with other KRAS-mutated tumors. Furthermore, in vitro experiments revealed that cancer cells with KRAS codon 13 mutation had a weaker level of resistance to apoptosis than those with KRAS codon 12 mutations [12]. However, these findings were obtained from retrospective studies or subgroup analyses. Therefore, a prospective validation study was warranted to confirm those findings. On the other hand, the randomized phase II BOND study (Cetuximab Monotherapy and Cetuximab plus Irinotecan in Irinotecan-Refractory Metastatic Colorectal Cancer) demonstrated significant improvement in response rate and progression-free survival with the addition of irinotecan to cetuximab in the refractory setting in molecularly unselected patients whose disease had progressed on irinotecan. According to the Bond study, we estimated synergy effects between cetuximab and irinotecan or response to irinotecan rechallenge despite prior progression on that drug.

To optimize mCRC patients’ treatment options, this randomized phase II study was designed with the aim of evaluating the efficacy of cetuximab and the additional effect of irinotecan on cetuximab-based therapy in mCRC patients with a mutation in KRAS codon G13D.

## Materials and methods

### Study design

This was a multicenter, open-label, prospective phase II study investigating the efficacy and safety of cetuximab and irinotecan plus cetuximab in patients with chemo-refractory mCRC with mutation at KRAS codon G13D. The study data and informed consent were obtained in accordance with the Declaration of Helsinki, and the study protocol was approved by the Ethics Review Board of each institution.

The study design was an observation-enriched randomized controlled design. During the informed consent process, each patient selected whether they wanted to join the observation arm or randomization arm if they accepted the trial participation. If patients chose the observation arm, they picked the study treatment (irinotecan alone or irinotecan plus cetuximab) they wanted to be in. Otherwise, they were centrally randomized to irinotecan alone or irinotecan plus cetuximab with a minimization technique (stratification factors were performance status and institution).

### Patients

Patients were eligible if they were ≥20 years of age and had histologically confirmed mCRC; already received first-line chemotherapy for mCRC; a known G13D KRAS mutation status; measurable disease as assessed by computed tomography or magnetic resonance imaging according to Response Evaluation Criteria in Solid Tumors (RECIST); an Eastern Cooperative Oncology Group performance status ≤1; and adequate hepatic, renal, and bone marrow function [white blood cell (WBC) count ≥3000 and ≤12,000/mm^3^, neutrophil count ≥1500/mm^3^, platelet count ≥100,000/mm^3^, GOT and GPT ≤100 U/l, total bilirubin <1.5 mg/dl, creatinine <1.5 mg/dl, and normal ECG]. Patients were ineligible if they had a history of previous exposure to EGFR-targeted therapy and/or uncontrolled severe organ/metabolic dysfunction.

### Study treatment

The enrolled patients started protocol treatment within 14 days after registration: a biweekly intravenous (IV) infusion of cetuximab (500 mg/m^2^ on day 1) or a weekly IV infusion of cetuximab (an initial intravenous infusion of 400 mg/m^2^ with subsequent weekly doses of 250 mg/m^2^) (Cet group); a biweekly IV infusion of irinotecan (150 mg/m^2^ on day 1) plus a biweekly IV infusion of cetuximab (500 mg/m^2^ on day 1) or a weekly IV infusion of cetuximab (an initial intravenous infusion of 400 mg/m^2^ with subsequent weekly doses of 250 mg/m^2^) (CetI group).

### KRAS and BRAF mutations

DNA was extracted from formalin-fixed paraffin-embedded tumor tissues. Mutation of KRAS at codon 13 was detected by direct sequencing, as described previously [13, 14].

### Endpoints

The primary endpoint was progression-free survival (PFS). The PFS of patients without disease progression before the end of the study was censored at the last on-study tumor assessment date at which the patient was considered to be progression-free. PFS was defined as the number of days between the enrollment and the first on-study assessment of disease progression (PD) or any cause of death.

The secondary endpoints were the disease control rate, overall survival (OS), and the safety of the combination therapy. The RECIST 1.1 criteria were applied subsequently to assess and confirm the overall response. A radiologic assessment was performed at baseline, every 8 weeks during the first 6 months of the study, and every 12 weeks thereafter until PD. The OS was defined as the number of days between the enrollment and any cause of death. Patients who did not die were censored at the last follow-up date. Adverse events were collected throughout the study period. All adverse events recorded were graded according to the Common Terminology Criteria for Adverse Events version 4.0.

### Statistical analyses

For the crude analysis, we analyzed all patients who enrolled in the trial without statistical adjustments. All summary statistics on time-to-event variables were calculated according to the Kaplan–Meier methods. The 95% confidence intervals (CIs) for median time-to-event and the time-specific incidence rate were constructed using Greenwood’s formula and Brookmeyer and Crowley’s method, respectively. The Cox proportional hazard model was used to estimate the hazard ratio (HR) and its 95% CI between the Cet group and CetI group.

Propensity score was used to adjust the unbalance of prognostic factor between Cet group and CetI group in the observation arm to combine results of the randomization arm with the observation arm. Propensity score was estimated using the logistic regression model. The variable used for the logistic model was selected based on the standardized difference between two groups. Using the stabilized inverse propensity score as weight, we created the pseudo-population using the total patients of observation arm as the standard population. A stratified analysis was used to summarize the results from randomization arm and observation arm.

The sample size was determined only for the randomization group. Assuming an absolute difference in 6 month PFS rate of ≥26% between the C group and CI group, 15 patients for each group were needed to observe improved treatment with 95% probability. We planned to enroll patients in the observation arm until the target sample sizes in the randomization arm were achieved.

## Results

### Patients’ characteristics

**Fig. 1:**
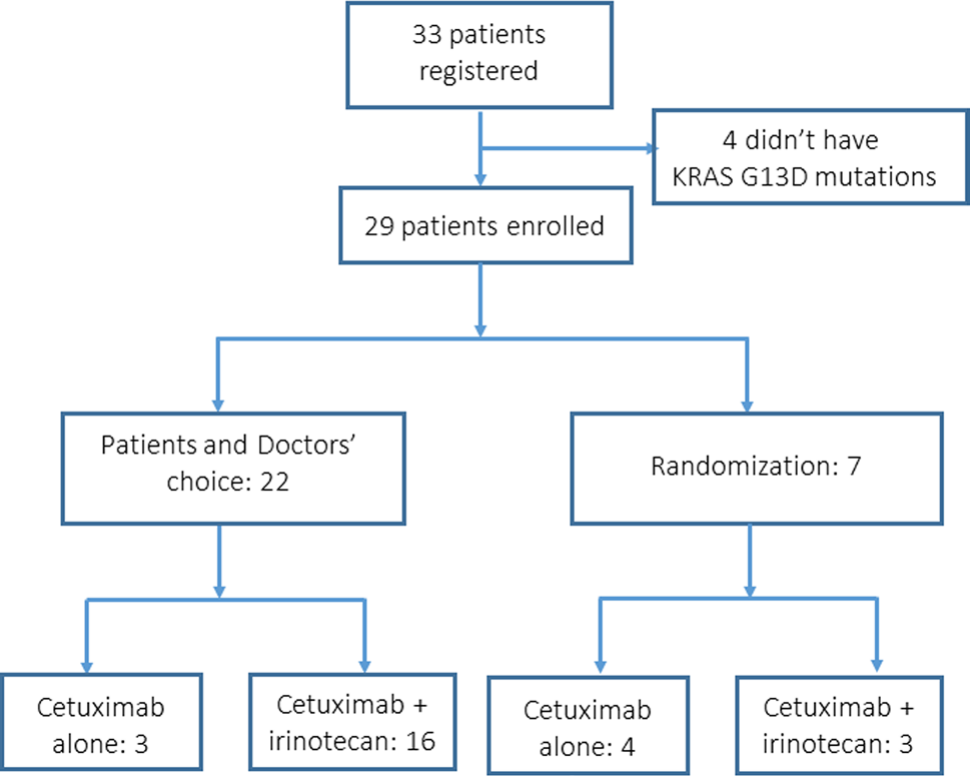
CONSORT diagram of the present study

From July 2012 to May 2015, 43 institutions collaborated with the G13D-study, and 33 patients were registered from 23 institutions. Four patients did not have KRAS G13D mutations. Accordingly, 29 patients were defined as eligible for this study. Figure 1 shows the CONSORT diagram. The intention-to-treat population consisted of 29 patients. A safety analysis was carried out on those 29 patients who received at least 1 dose of any component of the study treatment. The patients’ clinical characteristics at baseline are shown (Table 1). The median age of the patients was 69 years (range 37–83 years). In the first-line chemotherapy, 16 patients received bevacizumab and chemotherapy and 4 patients received irinotecan-based chemotherapy. Twelve patients (41.4%) had metastatic lesions in 1 organ, and 17 patients (58.6%) had metastatic lesions in more than 1 organ. As expected, hepatic metastases were the most common, with lesions detected in 17 patients (58.6%).

**Table 1:** Patient characteristics

Characteristics	Total (*n* = 29)	Cetuximab alone (*n* = 10)	Cetuximab + CPT-11 (*n* = 19)
	No. of patients (%)	No. of patients (%)	No. of patients (%)
Gender			
Male	21 (72.4)	8 (80.0)	13 (68.4)
Female	8 (27.6)	2 (22.2)	6 (31.6)
Age (years)			
Median	69	69	69
Range	(37–83)	(58–80)	(37–83)
ECOG performance status			
0	19 (65.5)	4 (40.0)	15 (78.9)
1	10 (34.5)	6 (60.0)	4 (21.1)
Cancer location			
Colon	16 (55.2)	7 (70.0)	9 (47.4)
Rectum	13 (44.8)	3 (30.0)	10 (52.6)
Primary site resection			
No	19 (65.5)	6 (60.0)	13 (68.4)
Yes	10 (34.5)	4 (40.0)	6 (31.6)
Previous chemotherapy therapy line			
1st, 2nd line	25 (86.2)	8 (80.0)	17 (89.5)
3rd, 4th line	4 (13.8)	2 (20.0)	2 (10.5)
No. of organs with metastases			
1	12 (41.4%)	4 (40.0)	8 (42.1)
2	9 (31.0%)	5 (50.0)	4 (21.1)
>3	8 (27.6%)	1 (10.0)	7 (36.8)
Metastases site			
Liver	17	5 (50.0)	12 (63.2)
Lung	16	7 (70.0)	9 (47.4)
Lymph node	10	3 (30.0)	7 (36.8)

*ECOG* Eastern Cooperative Oncology Group

### Efficacy

**Fig. 2:**
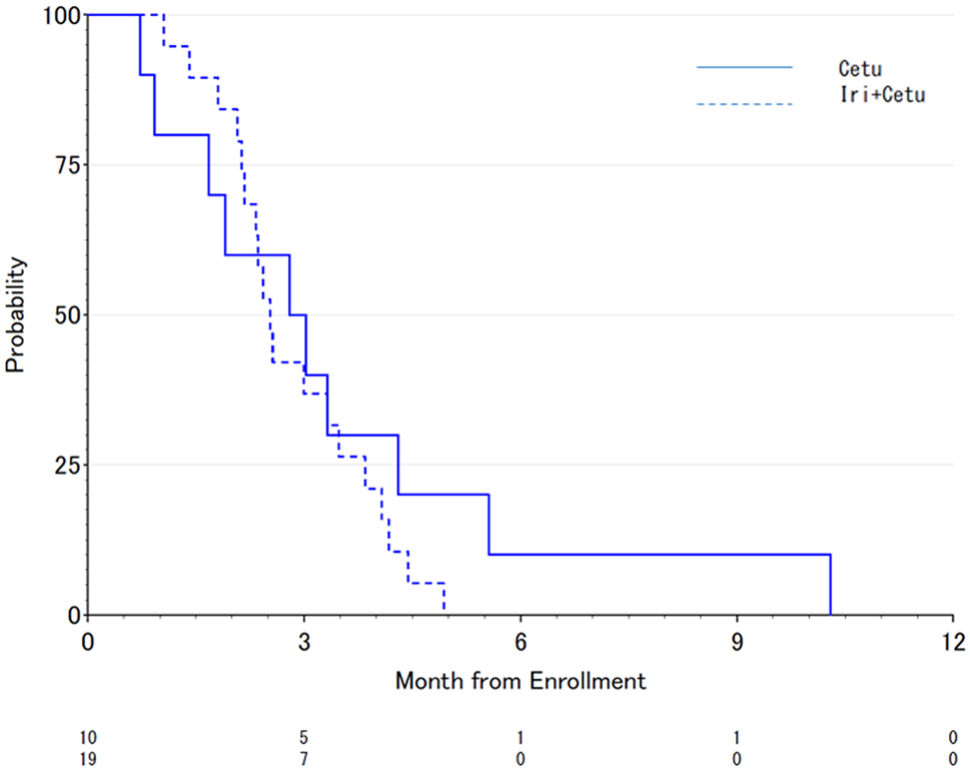
Progression-free survival in patients treated with cetuximab alone versus cetuximab + irinotecan

**Fig. 3:**
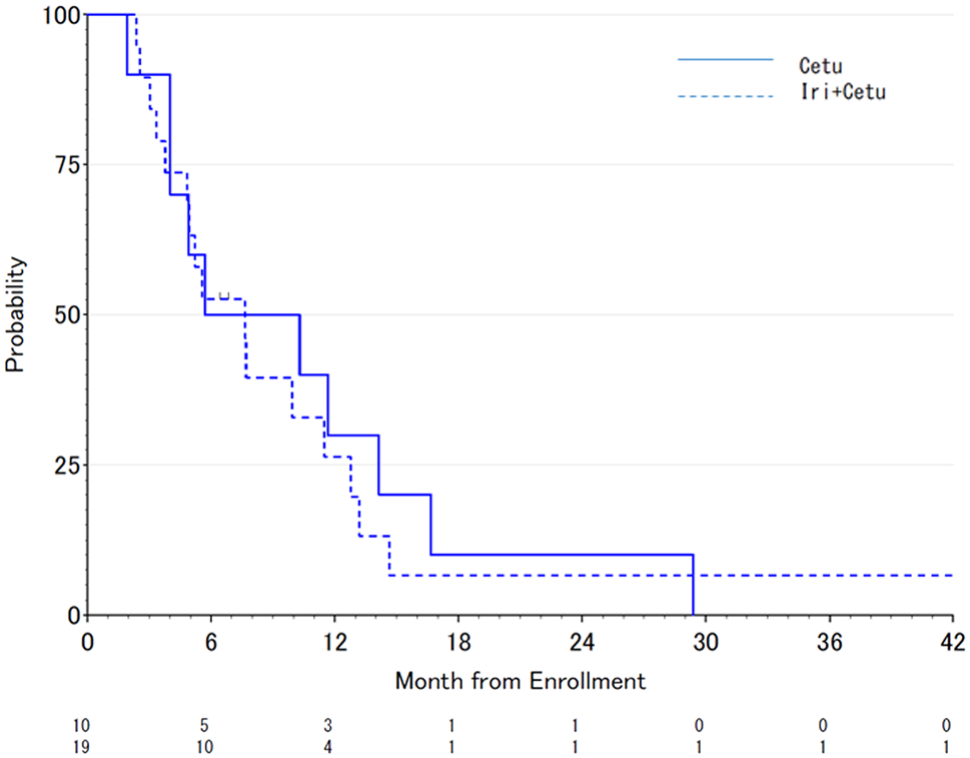
Overall survival in the patients treated with cetuximab alone versus cetuximab + irinotecan

After a median follow-up of 43 months, the crude median PFS was 2.9 months (95% CI 0.7–4.3) in the Cet group and 2.5 months (95% CI 2.1–3.5) in the CetI group (Fig. 2), which was not statically different (HR = 0.69, 95% CI 0.30–1.59, *p* = 0.380). In addition, the crude median OS was 8.0 months (95% CI 1.9–14.1) in the Cet group and 7.6 months (95% CI 3.9–11.5) in the CetI group (Fig. 3), which was not statically different (HR = 0.87, 95% CI 0.39–1.95, *p* = 0.741). In addition, 6-month progression-free survival rates were 10% (95% CI 0.5–35.8%) in Cet group and 0% in CetI group. There was not significantly difference between two groups (*p* = 0.375).

**Fig. 4:**
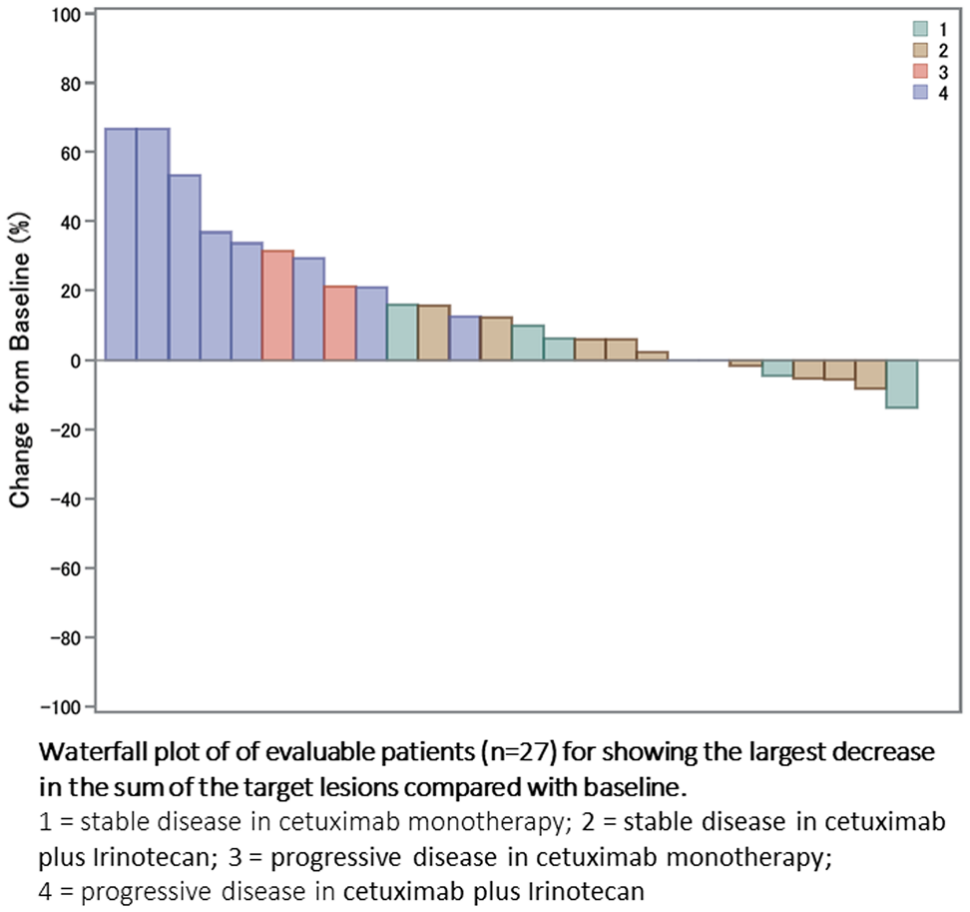
Best response of the present study

The efficacy data are summarized in Table 2. The disease control rate (DCR) was 60% (95% CI 26–88) in the Cet group and 47% (95% CI 24–71) in the CetI group, which was not statically different (*p* = 0.800). The best response is shown in Fig. 4. In addition, the DCR was 41% in the patients who had hepatic metastases (40% in the Cet group and 42% in the CetI group), 50% in the patients who had lung metastases (57% in the Cet group and 44% in the CetI group), and 50% in the patients who had lymphatic metastases (100% in the Cet group and 29% in the CetI group). There were no differences in the DCR by metastatic organ.

**Table 2:** Efficacy data

Parameter	All cases	Cetuximab alone (*n* = 10)	Cetuximab + CPT-11 (*n* = 19)	*p* value
Best overall response rate				
Complete response	0	0	0	
Partial response	0	0	0	
Stable disease	15	6	9	
Progressive disease	12	2	10	
Not assessable	2	2	0	
Disease control rate	51.7%	60.0%	47.4%	0.800
Median PFS (months)	2.6	2.9	2.5	0.380
95% CI	2.1–3.3	0.7–4.3	2.1–3.5	
Progression events	29	10	19	
Censored	0	0	0	
Median OS (months)	7.6	8.0	7.6	0.741
95% CI	4.8–11.5	1.9–14.1	3.9–11.5	
Deaths	26	10	16	
Censored	3	0	3	
1-year survival rate	27.9	30.0	26.3	
95% CI	9.9–41.1	7.1–57.8	8.6–48.4	
2-year survival rate	8.0	10	6.6	
95% CI	1.4–22.3	0.6–35.8	0.4–25.6	

*PFS* progression-free survival, *OS* overall survival, *CI* confidence interval

### Efficacy results after adjustment using propensity score

In the observation arm, performance status and number of metastasis showed discrepancy based on the standardized difference between Cet group and CetI group. Thus, we estimated propensity score using performance status and number of metastasis as covariates. After adjustment using propensity score, the HR for PFS was 1.15 (95% CI 0.45–2.93) and there was no statistical difference between Cet and CetI group (*p* = 0.765). OS was also similar between two groups (HR = 1.03; 95% CI 0.44–2.41; *p* = 0.950).

### Treatment compliance and safety

The median course of the study treatment was 2.6 months (range 2.2–3.4). The median course of the study treatment was 2.8 months (95% CI 0.7–4.7) in the Cet group and 2.5 months (95% CI 2.2–3.5) in the CetI group, which was not statically different. Patients achieving a relative dose intensity >80% for each drug are also shown.

Adverse events (AEs) of grade 3 or 4 occurring in at least 34.5% (10/29 patients) of the patients are summarized (Table 3). The most common grade 3 or 4 AEs were neutropenia, anorexia, and fatigue. Although the incidence of neutropenia was higher in the CetI group than in the Cet group, the rates of the other adverse events were similar between the two groups.

**Table 3:** Relevant adverse events

Adverse event	All cases (Grade 3/4)		Cetuximab alone (Grade 3/4)		Cetuximab + CPT-11 (Grade 3/4)	
	Number of patients (%)		Number of patients (%)		Number of patients (%)	
Hematological						
Leukopenia	1	3.4	0	0	1	5.3
Neutropenia	4	13.8	0	0	4	21.2
Anemia	2	6.8	1	10.0	1	5.3
Thrombocytopenia	0	0	0	0	0	0
No hematological						
Elevated ALP	2	6.8	1	10.0	1	5.3
Elevated serum Bil	0	0	0	0	0	0
Elevated creatine	0	0	0	0	0	0
Stomatitis	0	0	0	0	0	0
Nausea/vomiting	1	3.4	0	0	1	5.3
Diarrhea	1	3.4	0	0	1	5.3
Rash	0	0	0	0	0	0
Paronychia	0	0	0	0	0	0
Anorexia	4	13.8	2	20.0	2	10.6
Fatigue	4	13.8	1	10.0	3	15.9
Infusion related reaction	0	0	0	0	0	0
Paresthesia	0	0	0	0	0	0

*ALP* alkaline phosphatase, *Bil* bilirubin

## Discussion

This study was a prospective clinical trial to investigate the efficacy and safety of cetuximab-based treatment in patients with chemotherapy-resistant, refractory mCRC with KRAS G13D mutation. The present study showed that the treatment effects were similar to those in patients with KRAS wild-type tumors. Cetuximab-based treatment seemed to benefit patients with chemotherapy-resistant, refractory KRAS G13D-mutated mCRC, despite their mutation.

The median progression-free survival (PFS) was 2.9 months (95% CI 0.7–4.3) in the Cet group and 2.5 months (95% CI 2.1–3.5) in the CetI group in the present study. Similar PFS was observed, regardless of the treatment regimen. These results were similar to those of previous phase reports regarding the administration of cetuximab-based treatment in patients with refractory mCRC with KRAS G13D mutation. There have been two reports evaluating PFS in this setting. Bando et al. [15] retrospectively investigated the relationship between KRAS status and cetuximab efficacy among Japanese patients. In their study, 7 patients with refractory mCRC with KRAS G13D mutation were treated by a cetuximab + irinotecan regimen. No statistically significant differences in the PFS were observed between KRAS p.G13D-mutant and other mutant tumors. In addition, De Roock et al. [11] studied the association of KRAS G13D mutation with outcome after cetuximab treatment in a pooled data set of 579 patients with chemotherapy-refractory metastatic CRC treated with cetuximab with or without chemotherapy. They found that the patients with G13D-mutated tumors (*n* = 32) treated with cetuximab had longer PFS than the patients with KRAS-mutated tumors in other locations (median 4.0 [95% CI 1.9–6.2] months vs. 1.9 [95% CI 1.8–2.8] months; adjusted HR = 0.51 [95% CI 0.32–0.81; *p* = 0.004]).

The PFS in the present study was slightly shorter than in previous reports, possibly due to differences in the study design, patient backgrounds, and details of treatment. In contrast, there was no significant difference in the PFS between the Cet group and the CetI group in the present study (HR = 0.69, 95% CI 0.30–1.59, *p* = 0.380). Similar results were observed in a recent phase II study; Segelov et al. [16] assessed cetuximab monotherapy and cetuximab plus irinotecan in patients with molecularly selected (G13D mutation) chemotherapy-refractory mCRC in a randomized phase II trial (ICECREAM study). They found that PFS in patients with KRAS G13D-mutated mCRC did not significantly differ between those treated with cetuximab monotherapy and those treated with combined cetuximab and irinotecan. Moreover, when comparing the present study results and ICECREAM trial results, the 6-month PFS of cetuximab monotherapy was similar although the number of patients was small. In the present study, we estimated synergy effects between cetuximab and irinotecan or response to irinotecan rechallenge despite prior progression on that drug in this setting. However, these effects were not observed in the present study. However, De Roock et al. reported conflicting findings that the median PFS was 1.8 (95% CI 1.7–11.0) months in the cetuximab monotherapy group (*n* = 10) versus 4.1 (95% CI 2.8–6.9) months in the cetuximab plus chemotherapy group (*n* = 22). These results might suggest a synergetic effect of cytotoxic agents and targeted agents or persistent chemotherapy sensitivity [16]. Future studies should focus on this issue.

The crude median OS was 8.0 months (95% CI 1.9–14.1) in the Cet group and 7.6 months (95% CI 3.9–11.5) in the CetI group, which was not statically different (HR = 0.87, 95% CI 0.39–1.95, *p* = 0.741). A similar HR was observed in the ICECREAM study (HR = 0.95 [95% CI 0.53–1.68]). Furthermore, the disease control rate (DCR) was 60% in the Cet group and 47.4% in the CetI group. The ICECREAM study showed that the response and stable disease rates were 0 and 58% for cetuximab monotherapy and 9 and 70% for cetuximab + irinotecan combination treatment, respectively [16].

Although 35% of patients experienced grade 3/4 AE in our study and hematological toxicities were slightly higher with combination therapy, the incidence of the worse toxicities in the combination was within the previously reported ranges. The cetuximab-based treatment was generally well tolerated, and there was no evidence suggesting that cetuximab-based treatment increased the frequency or severity of characteristic toxicities. The rate of grade 3/4 AE was 44–59% in the ICECREAM study [16].

One important limitation of the present study is the lack of pertaining to comparison with outcomes of previous standard regimen. The standard second-line treatment was cytotoxic chemotherapy, such as FORFOX or FORFIRI, with/without bevacizumab typically used for refractory mCRC patients with mutations of KRAS in codon 13 mutation. Moreover, the standard third-line treatment was cytotoxic chemotherapy for refractory mCRC patients with mutations of KRAS in codon 13 mutation. However, the patient’s background and treatment were quite different between the present study and the previous studies. Therefore, it is difficult to draw the definitive conclusions from the present study.

In conclusion, cetuximab-based treatment seemed to benefit patients with chemotherapy-resistant, refractory mCRC with KRAS G13D mutation. In addition, the present study showed that the cetuximab-based treatment was well tolerated and had a manageable safety profile for chemo-refractory mCRC with KRAS G13D mutation. Our results might support the administration of cetuximab-based treatment for mCRC with KRAS mutation in codon G13D despite their mutation, providing treatment flexibility in this setting.
